# Associations Among Hair Cortisol Concentrations, Posttraumatic Stress Disorder Status, and Amygdala Reactivity to Negative Affective Stimuli in Female Police Officers

**DOI:** 10.1002/jts.22395

**Published:** 2019-03-18

**Authors:** Mirjam van Zuiden, Mesut Savas, Saskia B.J. Koch, Laura Nawijn, Sabine M. Staufenbiel, Jessie L. Frijling, Dick J. Veltman, Elisabeth F.C. van Rossum, Miranda Olff

**Affiliations:** ^1^ Department of Psychiatry Amsterdam University Medical Centers–Academic Medical Center University of Amsterdam Amsterdam the Netherlands; ^2^ Department of Internal Medicine, Division of Endocrinology, Erasmus Medical Center University Medical Center Rotterdam Rotterdam the Netherlands; ^3^ Donders Institute for Brain, Cognition and Behaviour Radboud University–Nijmegen Nijmegen the Netherlands; ^4^ Department of Psychiatry Amsterdam University Medical Centers–VU University Medical Center Amsterdam the Netherlands; ^5^ Arq Psychotrauma Expert Group Diemen the Netherlands

## Abstract

Posttraumatic stress disorder (PTSD) is associated with altered hypothalamic‐pituitary‐adrenal (HPA) axis function. Measurement of hair cortisol concentrations (HCC) allows retrospective assessment of HPA axis regulation over prolonged periods of time. Currently, research investigating HCC in PTSD remains sparse. Previous cross‐sectional studies have included only civilian populations, although it is known that trauma type moderates associations between PTSD status and HPA axis function. We investigated differences in HCC between trauma‐exposed female police officers with current PTSD (*n* = 13) and without current and lifetime PTSD (*n* = 15). To investigate whether HCC was associated with neural correlates of PTSD, we additionally performed exploratory correlational analyses between HCC and amygdala reactivity to negative affective stimuli. We observed significantly lower HCC in participants with PTSD than in participants without PTSD, *d* = 0.89. Additionally, within participants with PTSD, we observed positive correlations between HCC and right amygdala reactivity to negative affective (vs. happy/neutral) faces, *r* = .806 (*n* = 11) and left amygdala reactivity to negative affective (vs. neutral) pictures, *r* = .663 (*n* = 10). Additionally, left amygdala reactivity to negative faces was positively correlated with HCC in trauma‐exposed controls, *r* = .582 (*n* = 13). This indicates that lower HCC is associated with diminished amygdala differentiation between negative affective and neutral stimuli. Thus, we observed lower HCC in trauma‐exposed noncivilian women with PTSD compared to those without PTSD, which likely reflects prolonged HPA axis dysregulation. Additionally, HCC was associated with hallmark neurobiological correlates of PTSD, providing additional insights into pathophysiological processes in PTSD.

## Resumen

Spanish Abstracts by Asociación Chilena de Estrés Traumático (ACET)

**Concentraciones de cortisol en cabello se asocian con la condición de TEPT y la reactividad de la amígdala a estímulos emocionales negativos en oficiales de policía mujeres**

BAJO NIVEL DE CORTISOL EN CABELLO EN PACIENTES MUJERES CON TEPT

El trastorno de estrés postraumático (TEPT) está asociado con la función alterada del eje hipotálamo‐hipófisis‐adrenal (HPA). La medición de las concentraciones de cortisol en cabello (HCC por sus siglas en inglés) permite evaluar en forma retrospectiva la regulación del eje HPA en periodos prolongados. Actualmente las investigaciones de HCC en TEPT son escasas. Los estudios transversales previos solo han incluido población civil, aunque se sabe que el tipo de trauma modera las asociaciones entre la condición del TEPT y la función del eje HPA. Investigamos las diferencias en el HCC entre oficiales de policía de sexo femenino expuestos a trauma con TEPT actual (*n* = 13) y sin TEPT actual o a lo largo de su vida (*n* = 15). Para investigar si la HCC se asociaba con correlatos neurales del TEPT, adicionalmente realizamos un análisis exploratorio correlacional entre la HCC y reactividad de la amígdala a estímulos emocionales negativos. Observamos niveles significativamente más bajos de HCC en las participantes con TEPT que en las sin TEPT, *d* = 0.89. Adicionalmente, entre las participantes con TEPT, observamos correlaciones positivas entre HCC y la reactividad de la amígdala derecha a caras con emociones negativas (vs. felicidad/neutral), *r* = .806 (*n* = 11) y la reactividad de la amígdala izquierda a fotografías con emociones negativas (vs. Neutral) *r* = .663 (*n* = 10). Adicionalmente, la reactividad de la amígdala izquierda a caras con emociones negativas estuvo correlacionada positivamente con HCC en controles expuestos a trauma, *r* = .582 (*n* = 13). Esto indica que HCC más bajos se asocian con capacidad de diferenciación disminuida de la amígdala entre estímulos emocionales negativos y neutrales. Así, observamos niveles más bajos de HCC en mujeres no civiles expuestas a trauma con TEPT comparadas con aquellas sin TEPT, lo que probablemente refleja una prolongada desregulación del eje HPA. Adicionalmente, HCC se asoció con correlatos neurobiológicos distintivos del TEPT, proveyendo información adicional de los procesos patofisiológicos en el TEPT.

## 抽象

Traditional and Simplified Chinese Abstracts by the Asian Society for Traumatic Stress Studies (AsianSTSS)

簡體及繁體中文撮要由亞洲創傷心理研究學會翻譯

Hair Cortisol Concentrations are associated with PTSD Status and Amygdala Reactivity to Negative Affective Stimuli in Female Police Officers

Traditional Chinese

標題: 女警中, 毛髮中的皮質醇含量跟PTSD狀態和杏仁體對負面情感刺激的反應有關

撮要: 創傷後壓力症(PTSD)跟下丘腦‐垂體‐腎上腺軸(HPA)功能改變有關。在一段長時間裡, 測量毛髮中的皮質醇含量(HCC), 可對HPA軸的調節作回顧性評估。目前對於PTSD患者的HCC研究仍然很少。即使我們知道創傷類別對PTSD狀態和HPA軸的關連有調節作用, 過往的橫斷研究也只包含平民為樣本。本研究檢視有當前PTSD的受創女警(n = 13)和沒有當前和終生PTSD的女警(n = 15)之間HCC的差異。為探查HCC是否跟PTSD的神經關連因素相關, 我們額外對HCC以及杏仁體對負面情感刺激的反應進行探索性關連分析。相比無患PTSD的樣本, 患PTSD的樣本有顯著較低的HCC(d = 0.89)。此外, 患PTSD的樣本中, HCC跟右邊杏仁體對負面情感表情 (比對快樂/中性表情) 的反應有正向關連, r = .806 (n = 11), 亦跟左邊杏仁體對產生負面情感的圖片 (比對中性圖片) 的反應有正向關連, r = .663 (n = 10)。另外, 在受創的對照樣本中, 左邊杏仁體對負面表情的反應跟HCC有正向關連, r = .582 (n = 13)。結果反映, HCC較低跟杏仁體對負面情感和中性刺激的辨別能力減弱有關。因此, 相比無患PTSD的受創非平民女性, 患PTSD的有較低的HCC, 而那很可能反映有長期的HPA軸失調。HCC亦跟PTSD的神經生理關連因素特徵有關。研究結果加深了我們對PTSD的病理生理學過程的理解。

标题: 女警中, 毛发中的皮质醇含量跟PTSD状态和杏仁体对负面情感刺激的反应有关

撮要: 创伤后压力症(PTSD)跟下丘脑‐垂体‐肾上腺轴(HPA)功能改变有关。在一段长时间里, 测量毛发中的皮质醇含量(HCC), 可对HPA轴的调节作回顾性评估。目前对于PTSD患者的HCC研究仍然很少。即使我们知道创伤类别对PTSD状态和HPA轴的关连有调节作用, 过往的横断研究也只包含平民为样本。本研究检视有当前PTSD的受创女警(n = 13)和没有当前和终生PTSD的女警(n = 15)之间HCC的差异。为探查HCC是否跟PTSD的神经关连因素相关, 我们额外对HCC以及杏仁体对负面情感刺激的反应进行探索性关连分析。相比无患PTSD的样本, 患PTSD的样本有显著较低的HCC(d = 0.89)。此外, 患PTSD的样本中, HCC跟右边杏仁体对负面情感表情 (比对快乐/中性表情) 的反应有正向关连, r = .806 (n = 11), 亦跟左边杏仁体对产生负面情感的图片 (比对中性图片) 的反应有正向关连, r = .663 (n = 10)。另外, 在受创的对照样本中, 左边杏仁体对负面表情的反应跟HCC有正向关连, r = .582 (n = 13)。结果反映, HCC较低跟杏仁体对负面情感和中性刺激的辨别能力减弱有关。因此, 相比无患PTSD的受创非平民女性, 患PTSD的有较低的HCC, 而那很可能反映有长期的HPA轴失调。HCC亦跟PTSD的神经生理关连因素特征有关。研究结果加深了我们对PTSD的病理生理学过程的理解。

Posttraumatic stress disorder (PTSD) is associated with altered hypothalamic‐pituitary‐adrenal (HPA) axis function; this has mainly been investigated by assessing acute cortisol levels and glucocorticoid receptor (GR) function (Olff & van Zuiden, 2017). Insight into chronic HPA axis dysregulation may be gained by assessing cortisol in scalp hair, which allows retrospective assessment over prolonged periods of time (Staufenbiel, Penninx, Spijker, Elzinga, & van Rossum, 2013). Research on hair cortisol concentrations (HCC) in PTSD has been relatively sparse. In one cross‐sectional study, higher levels of HCC were observed in individuals with PTSD compared to trauma‐exposed controls (TCs) in a mixed‐gender sample of refugees with ongoing distress (Steudte et al., 2011). In another study, the authors found lower levels of HCC in trauma‐exposed individuals with and without PTSD compared to non‐trauma‐exposed controls in a predominantly female sample (Steudte et al., 2013). Additionally, no HCC differences were found between female refugees with stress‐related disorders and TCs (Schalinski, Elbert, Steudte‐Schmiedgen, & Kirschbaum, 2015) or between male refugees who had recently fled their country of origin with and without PTSD (Mewes, Reich, Skoluda, Seele, & Nater, 2017). In a longitudinal study, pretrauma HCC did not differ between female adolescent earthquake survivors with and without PTSD at 7 months after the earthquake (Luo et al., 2012). Acute posttrauma HCC was elevated in both groups but had normalized at 5–7 months after the earthquake in TCs. At the final assessments, survivors with subsequent PTSD had lower HCC than TCs (Luo et al., 2012). In a prospective study, high HCC in the acute posttrauma period predicted subsequent PTSD symptoms in a predominantly female sample of individuals hospitalized with physical injuries (Pacella, Hruska, Steudte‐Schmiedgen, George, & Delahanty, 2017). In the only study to our knowledge that has used a noncivilian sample, low predeployment HCC predicted PTSD symptom development in male soldiers upon trauma exposure during deployment (Steudte‐Schmiedgen et al., 2015). Trauma type has been shown to moderate the association between PTSD and acute cortisol levels (Meewisse, Reitsma, de Vries, Gersons, & Olff, 2007). To date, associations between HCC and PTSD have been predominantly investigated in female civilian populations. It remains unknown whether previous findings extend to noncivilian populations, particularly those at risk for cumulative exposure to work‐related traumatic events.

To understand the pathophysiology and clinical and phenotypic heterogeneity of PTSD, it is of interest to investigate whether chronic HPA axis dysregulation is associated with neural correlates of PTSD. Glucocorticoid receptor and mineralocorticoid receptor (MR) activation affect amygdala excitability and synaptic connectivity and may induce anxiogenic responses, with exact effects depending on duration, timing, and amount of current and prior glucocorticoid exposure (de Quervain, Schwabe, & Roozendaal, 2017). This is of relevance for PTSD as several meta‐analyses have observed significant amygdala hyperreactivity in individuals with PTSD (Hayes, Hayes, & Mikedis, 2012; Koch et al., 2016a; Patel, Spreng, Shin, & Girard, 2012; Sartory et al., 2013; Stark et al., 2015). This fits with the predominant neurocircuitry model for PTSD, which posits a central role for increased salience and threat hypersensitivity by brain areas within the salience network, including the amygdala, that is paralleled by inadequate top‐down regulation by ventromedial prefrontal areas (Koch et al., 2016a). In these meta‐analyses, specifically those related to right amygdala hyperreactivity in response to non‐trauma‐related negative stimuli (Stark et al., 2015), differences in amygdala functioning between trauma‐exposed individuals with and without PTSD were observed, whereas no differences in amygdala reactivity to trauma‐related stimuli were observed between PTSD and TC groups (Sartory et al., 2013).

In the current study, we investigated HCC differences between trauma‐exposed female police officers with (*n* = 13) and without PTSD (*n* = 15). We additionally performed exploratory analyses to assess associations between HCC and amygdala reactivity to non‐trauma‐related negative affective stimuli and a mixture of non‐trauma‐related and potentially trauma‐related negative affective stimuli. The current study comprised a subsample of a larger neuroimaging study that included both women and men. In the larger study, we did not observe significant group differences in amygdala reactivity toward negative affective stimuli (Koch et al., 2016b). However, although TCs showed increased amygdala reactivity to negative compared to neutral or positive affective faces, this differential reactivity was absent in participants with PTSD, indicating there may be increased saliency attributed to potential affective aspects of faces (Koch et al., 2016b).

## Method

### Participants

This study was part of a randomized controlled trial on neural effects of a single oxytocin administration in male and female trauma‐exposed police officers with and without PTSD who were between 18 and 65 years of age (Koch et al., 2016b). Data presented in the current manuscript were collected at baseline or during the session in which they received a placebo. For the current study, we included female participants only, as the overlarge majority of male participants did not have sufficient hair length for HCC determination. Hair segments were available for 14 participants with PTSD and 16 TCs. One participant with PTSD and one TC were excluded due to extreme HCC values (described later), which resulted in 13 patients with PTSD and 15 TCs. Additionally, two patients with PTSD dropped out of the study prior to the placebo‐scanning session, and one additional participant with PTSD did not complete the picture task during this session. Thus, for participants with PTSD, imaging data were available for 11 participants for the faces task and 10 for the pictures task (tasks are described later in this article). Imaging data were available for all TCs, but one TC was excluded due to a scanning artefact in the temporal cortex.

Participants with PTSD fulfilled criteria given in the fourth edition of the *Diagnostic and Statistical Manual of Mental Disorders* (*DSM‐IV*) for current PTSD, with a total score on the Clinician‐Administered PTSD Scale for *DSM‐IV* (CAPS) of 45 or higher (Blake et al., 1995). Exclusion criteria for participants with PTSD were current severe major depressive disorder (MDD) with psychotic symptoms and/or suicidal intent, suicidal ideation, alcohol/substance abuse (except smoking), bipolar disorder, and psychotic disorder. Individuals in the TC group had to have reported at least one *DSM‐IV* Criterion A traumatic event and scored a 15 or less on the CAPS. They were matched to patients based on sex, age, education, and years of service. Exclusion criteria for TCs were lifetime MDD or PTSD or any current *DSM‐IV* Axis I psychiatric disorder. Exclusion criteria for all participants were daily use of psychoactive medication (incidental use was allowed as long as it had not occurred less than 24 hr prior to scanning) or systemic glucocorticoids, serious medical conditions, a history of neurological disorders, and several common contraindications for magnetic resonance imaging (MRI) and oxytocin administration (Koch et al., 2016b).

We did not observe significant group differences in demographic or health characteristics (Table 1). There were 12 TCs (80.0%) and six participants with PTSD (50.0%) who were in active police duty, Fisher's exact test = 0.13, *p* = .127; this likely explained why TCs reported nominally more types of work‐related traumatic events than participants with PTSD, *t*(25) = 1.82, *p* = .080. Hair color differed between groups—the majority of participants with PTSD had brown hair whereas more participants in the TC group had blond hair, Fisher's exact test = 7.56, *p* = .045.

**Table 1:** Demographic, Health, Trauma History and Symptom Severity Characteristics for Female Trauma‐Exposed Police Officers With and Without Posttraumatic Stress Disorder (PTSD)

	PTSD (*n* = 13)				Trauma‐Exposed Controls (*n* = 15)				
Characteristic	*M*	*SD*	*n*	%	*M*	*SD*	*n*	%	*p*
Age (years)	42.00	7.96			38.00	9.98			.257^a^
Years of police service	15.88	9.91			18.73	8.13			.795^a^
Current active executive duty			6	50.0			12	80.0	.127^d^
PLES total score	12.92	8.38			18.73	8.13			.080^a^
ETI‐SF total score	5.42	5.90			4.40	5.49			.648^b^
CAPS total score	69.38	10.91			4.00	4.60			< .001^a^
Major depressive disorder			4	30.8			0	0.0	.035^d^
Body mass index (kg/m^2^)	25.00	4.14			26.43	3.26			.321^a^
Current smoker			3	25.0			5	33.3	.696^d^
AUDIT total score	3.17	4.13			3.60	1.64			.712^a^
Hormonal contraceptive use			5	41.7			7	46.7	1.000^c^
Local glucocorticoid use			2	15.4			0	0.0	.206^d^
Hair color									.045^d^
Black			1	7.7			0	0.0	
Brown			8	61.5			4	26.7	
Blond			3	23.1			9	60.0	
Grey			1	7.7			0	0.0	
Red			0	0.0			2	13.3	
Hair washing frequency									.105^d^
0–2 times per week			2	15.4			4	26.7	
3–4 times per week			7	53.8			2	13.3	
> 4 times per week			4	30.8			9	60.0	
Hair treatment within past 3 months^e^			7	53.8			7	46.7	.705^c^
Days between hair collection and scanning session	3.09	7.33			−0.33	15.24			.443^b^
Scanning session time of day (hh:mm)	14:09	1:38			15:12	2:05			.185^a^

*Note*. PLES = Police Life Events Scale; ETI = Early Trauma Inventory–Self‐report form; AUDIT = Alcohol Use Disorder Identification Test; CAPS = Clinician‐Administered PTSD Scale for *DSM‐IV*.

a Independent samples *t* test used.

b Mann‐Whitney *U* test used.

c Chi‐square test used.

d Fisher's exact test used.

e Includes coloration, bleaching, and/or permanent wave.

### Procedure

Participants were recruited through a diagnostic outpatient center for police personnel (PDC; Diemen, the Netherlands; *n* = 3 participants with PTSD) and advertisements (*n* = 11 participants with PTSD and all TCs). All participants provided verbal and written informed consent prior to study initiation. At baseline, inclusion and exclusion criteria were assessed using diagnostic clinical interviews and self‐report questionnaires. For patients recruited via the diagnostic outpatient center, clinical interviews administered during intake were used. After inclusion, participants completed two scanning sessions (described later). Participants were asked to abstain from alcohol and drugs 24 hr before scanning and from rigorous exercise, beverages except water, and nicotine for 2.5 hr before scanning. Prior to scanning, intranasal oxytocin (40 IU) and a placebo (0.9% saline) were administered in a randomized double‐blind crossover design. For this study, we only included imaging data collected under placebo. For most participants (i.e., 10 participants with PTSD who completed scanning, 90.1%; and 11 TCs, 73.3%), scalp hair for HCC determination was collected before trial medication administration. We found that HCC did not significantly differ between TCs whose samples were collected prior to versus after trial medication administration, *t*(13) =−0.81, *p* = .431. The average time in days between hair collection and placebo scan did not significantly differ between groups (Table 1). The study was approved by the Institutional Review Board of the Amsterdam University Medical Centers, location Academic Medical Center, Amsterdam, the Netherlands, and was registered in the Netherlands Trial Registry (NTR3516).

### Measures

#### PTSD symptoms

All participants with PTSD were originally diagnosed by a licensed clinician prior to study inclusion. For all participants, current PTSD symptom severity was assessed using the validated Dutch version of the CAPS (Blake et al., 1995; Hovens et al., 1994). The CAPS is the gold standard structured clinical interview for diagnosing PTSD and assessing PTSD symptom severity according to *DSM‐IV* criteria. It assesses the three symptom clusters: reexperiencing (five items, with a possible score range of 0–40), avoidance (seven items, with a possible score range of 0–56), and hyperarousal (five items, with a possible score range of 0–40), and distinguishes between estimated symptom frequency (score range: 0–4) and intensity (score range: 0–4) in the previous month. We calculated a total symptom severity score by summing the intensity and frequency scores for all items. Higher scores indicate a higher level of symptom severity in the past month. The Cronbach's alpha value for internal consistency for all items was high, Cronbach's α = .98. As stated, participants met inclusion criteria for the PTSD group if their total score was 45 or higher; this cutoff was used to ensure current symptom severity above the clinical threshold. This cutoff has high sensitivity for PTSD diagnosis (Weathers, Ruscio, & Keane, 1999).

#### Other Axis I disorders

Dutch versions of the Mini International Neuropsychiatric Interview (M.I.N.I; Sheehan et al., 1998) or the Structured Clinical Interview for *DSM‐IV* (SCID; First, Spitzer, Gibbon, & Williams, 2002) were used to assess other *DSM‐IV* Axis I psychiatric disorders (the SCID was used for patients recruited through the diagnostic center). Both structured clinical interviews are widely used, valid, and reliable for diagnosing current and lifetime psychiatric disorders (Lobbestael, Leurgans, & Arntz, 2011; Sheehan et al., 1998).

#### Work‐related trauma exposure

The 42‐item Dutch Police Life Events Checklist (PLES) was used to assess the number of different police work–related traumatic events participants had encountered (Carlier & Gersons, 1992). A total score was calculated using the first 41 items, each of which inquired about a different event, by summing the number of endorsed items (possible score range: 0–41).

#### Childhood trauma exposure

The Dutch translation of the short self‐report version of the Early Trauma Inventory (ETI‐SF) was used to assess trauma exposure during childhood (Bremner, Bolus, & Mayer, 2007; Rademaker, Vermetten, Geuze, Muilwijk, & Kleber, 2008). This is a valid and reliable measure of childhood trauma (Bremner et al., 2007). The questionnaire consists of 21 items that assess whether participants were exposed to different types of physical (nine items), sexual (six items), and emotional abuse (five items) as well as general traumas (11 items) before 18 years of age. We calculated a total score by summing the number of endorsed items (range: 0–21).

#### Alcohol abuse

The Dutch translation of the validated Alcohol Use Disorder Identification test (AUDIT; Bush, 1998) was used to assess current alcohol use and level of alcohol‐related risk. It contains 10 items that assess alcohol consumption and indicators of dependence and harmful drinking. A total score was calculated by summing all item scores (range: 0–40). In the current sample, Cronbach's alpha for all items was questionable, α = .64, presumably because all participants except one obtained the lowest possible scores for all seven items inquiring about dependence and harmful drinking whereas scores on the three items regarding consumption quantity varied. Additionally, participants self‐reported demographic characteristics, current active police duty (executive function), weight, and height to calculate body mass index, current smoking status, medication and hormonal contraceptive use, and hair characteristics (color, washing frequency, coloration, bleaching, and permanent wave application in past 3 months).

#### Hair cortisol assessment

Hair was collected from the posterior vertex. Upon collection, samples were taped to paper and stored in closed envelopes at room temperature. We assessed cortisol concentrations in the most proximal 3 cm of scalp hair, covering HCC in the 3 months before sample collection, using a validated protocol (Manenschijn, Koper, Lamberts, and van Rossum, 2011). Samples were cut, weighted, and incubated with 1.0 mL methanol for 16 hr at 52 °C. Then, methanol solutions containing cortisol extracts were transferred to new vials and evaporated under a nitrogen stream. After dissolving dried contents with 250 μl phosphate buffered saline (PBS), HCC were quantified with enzyme‐linked immunosorbent assay (ELISA; DRG Instruments GmbH, Marburg, Germany) following the manufacturer's protocol. The previously determined lower‐end detection limit for this assay is 1.5 nmol/l. The upper detection limit according to the manufacturer's protocol is 220.69 nmol/l. All measurements were performed in duplicate in one assay. The intra‐assay variability for internal controls was on average 1.3% (range: 0.4%–2.3%). As reported by the manufacturer, the assay cross‐reactivity with other steroid hormones is corticosterone (29.0%), cortisone (3.0%), 11‐deoxycortisol (less than 1.0%), 17‐OH progesterone (less than 0.5%), testosterone (less than 0.1%), and estradiol (less than 0.1%). As is standard in steroid hormone hair analysis, HCC were converted to pg/mg, taking the weight of the hair samples into account (*M* = 15.1 mg, *SD* = 11.7, *Mdn* = 12.0, range: 5.77–67.93 mg). Hair weight and final HCC were not significantly correlated, *r* = −.06, *p* = .754. Two samples were excluded from all analyses due to nondetectable (in one participant with PTSD) and extremely high (in one TC; HCC: 180.73 pg/mg, standardized *z* score = 4.86) HCC.

#### Functional MRI (fMRI)

Structural and functional MRI images were acquired with a 32‐channel head coil on a 3T Philips (Andover, MA) Achieva MR system. During the two scanning sessions, we presented two versions of each task, including different stimuli, in randomized counterbalanced order. Scanning sessions were scheduled in the afternoon or early evening (for more details concerning fMRI data acquisition, see Koch et al., 2016b). Amygdala reactivity to negative affective pictures was assessed using a distraction task with three conditions: (a) passive viewing of 20 neutral pictures, (b) passive viewing of 20 negative affective pictures, and (c) working memory performance during presentation of 20 negative affective pictures (McRae et al., 2010). Pictures were presented using an event‐related design with pseudorandom order for trial type. All trials were separated by an intertrial interval, which consisted of a fixation cross presented for 2000 ms. In the current study, we used data collected during the two passive viewing conditions. Pictures were selected from the International Affective Picture System (IAPS), based on normative valence and arousal ratings (Lang, Bradley, & Cuthbert, 2008). Pictures in the task versions were matched for normative valence, arousal, complexity, and luminescence. Negative pictures included scenes related to events police officers may encounter in their line of work (e.g., violence, accidents) and more general aversive scenes (e.g., malnourished children, war‐related scenes; Koch et al., 2018).

Amygdala reactivity to negative affective faces was assessed using an emotional face‐matching task that contained three conditions: (a) angry‐fearful faces, (b) neutral‐happy faces, and (c) scrambled faces (visuomotor control; Hariri, Tessitore, Mattay, Fera, & Weinberger, 2002). Each trial consisted of three stimuli, with a cue stimulus presented on top and two target stimuli presented below. Participants were instructed to match the emotional expression (emotional condition) or the orientation (visuomotor control) of the cue stimulus with one of the target stimuli. Faces were selected from the NimStim face stimuli set (see Koch et al., 2016b, for more details).

### Data Analysis

Functional MRI data were analyzed using SPM8. Preprocessing steps included realignment, slice‐time correction, coregistration, normalization to the Montreal Neurological Institute (MNI) template, and smoothing (faces: 5 mm full‐width half maximum [FWHM] kernel; pictures: 6 mm FWHM kernel, mirroring primary analyses in the larger study). At first level, the six realignment parameters were included, images were high‐pass filtered, and temporal autocorrelation was removed with the AR(1) process (Koch et al., 2016b). One TC was excluded due to a scanner artifact in the temporal cortex. Two participants with PTSD did not complete the placebo scanning session. Additionally, one participant with PTSD did not complete the pictures task.

For the affective pictures, we only used first‐level contrast images, which were obtained by subtracting amygdala reactivity to passive viewing of neutral pictures from reactivity to passive viewing of negative pictures (negative > neutral). Individual contrast estimates were extracted from 5 mm spheres surrounding left, xyz = −24, −8, −20, and right, xyz = 20, −6, −14, amygdala peak task activation within the region of interest (ROI) anatomical mask (Harvard–Oxford 50% probabilistic atlas) across participants in the larger study during the placebo session (whole‐brain family‐wise error corrected, *p*_FWE_ < .05; Koch et al, 2018).

For the affective faces, contrast images were obtained by subtracting amygdala reactivity during the control condition from reactivity to angry‐fearful faces (angry‐fearful > control) and happy‐neutral faces (happy‐neutral > control). Individual contrast estimates were extracted from 5 mm spheres surrounding left, xyz = −20, −8, −16, and right, xyz = 24, −10, −14, amygdala peak task activation within the ROI anatomical mask (Harvard–Oxford 50% probabilistic atlas) under placebo across participants and emotion conditions in the larger study (all *p*_FWE_ < .05). For the purpose of comparing results with results from amygdala reactivity to affective pictures, contrast estimates for amygdala reactivity toward neutral‐happy faces (vs. control) were subtracted from contrast estimates for amygdala reactivity toward angry‐fearful faces (vs. control; angry‐fearful > happy‐neutral).

Subsequent analyses were performed in SPSS (Version 24). We investigated whether data were normally distributed and contained outliers, standardized *z* score > |3.29|. Aside from the one removed extreme HCC value, no outliers were removed. Questionnaire data other than hair characteristics and medication use were missing for one participant with PTSD. Participants with missing data were excluded from analyses pairwise. Group differences in participant characteristics were assessed with independent sample *t* tests (normally distributed continuous variables); Mann‐Whitney *U* tests (nonnormally distributed continuous variables); chi‐square tests (categorical variables with cell frequencies of 5 or above), or Fisher's exact tests (categorical variables with cell frequencies less than 5). Group difference in (normally distributed) HCC was first assessed using an independent sample *t* test and Cohen's *d* effect size (representing the standardized difference between group means, with *d* = 0.2, *d* = 0.5, and *d* = 0.8 commonly interpreted as small, medium, and large effects, respectively (Cohen, 1977), followed by analyses of covariance (ANCOVA)s to control for potential confounders. To minimize the influence of included covariates on calculated effect size, generalized eta squared (ηG^2^) was calculated, reflecting the amount of variance in HCC explained by PTSD versus TC status. As a benchmark, ηG^2^ = .01, ηG^2^ = .06, and ηG^2^ = .14 can be interpreted as small, medium, and large effects, respectively (Olejnik & Algina, 2003).

Exploratory correlation analyses (Pearson's *r* for normally distributed variables; Spearman's rho for nonnormally distributed variables) were performed to investigate associations between HCC and amygdala reactivity and negative affective stimuli within the PTSD and TC groups separately. To test whether correlation coefficients significantly differed between groups, we applied Fisher's *z* tests to compare correlation coefficients for both groups (Diedenhofen & Musch, 2015). Partial correlations were performed to investigate potential confounding influences of age and daytime of scanning. Additionally, we investigated correlations with PTSD symptom severity in participants with PTSD only due to selected low symptom severity in TCs. We considered *p* values less than .050 (two‐sided) to be statistically significant. Data are expressed as means and standard deviations for continuous variables and absolute frequency and relative percentage for categorical variables.

## Results

### Group Differences in HCC

**Figure 1:**
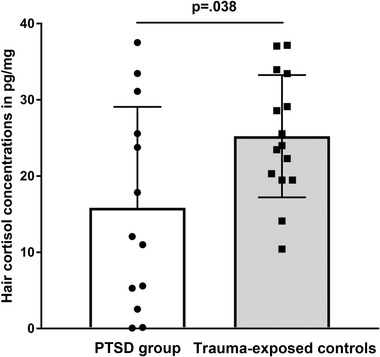
Hair cortisol concentrations (HCC) in 3 cm–long hair strands taken from the scalp of trauma‐exposed female police officers with *(n* = 13, left) and without *(n* = 15, right) posttraumatic stress disorder (PTSD). Points depict HCC values for each individual participant. Descriptive characteristics for each group are depicted as means (bars) and standard deviations (error bars).

Participants with PTSD had significantly lower HCC levels (*M* = 15.85 pg/mg, *SD* = 13.23) than trauma‐exposed participants without PTSD (*M* = 25.23 pg/mg, *SD* = 8.01), *t*(19.179) = 2.227, *p* = . 038, 95% CI [0.57, 18.19] (Figure 1). Cohen's *d* was 0.86, indicating a large effect size. This difference remained significant after controlling for work‐related traumatic events, *F*(1, 24) = 8.00, *p* = .009, ηG^2^ (i.e., explained variance in HCC by PTSD status) = .248, estimated *t*(24) = 2.83, 95% CI [3.20, 20.46]; and current active executive police duty, *F*(1, 24) = 5.84, *p* = .024, ηG^2^ = .189, estimated *t*(24) = 2.42, 95% CI [1.44, – 18.22]. Additionally, the difference remained significant after controlling for hair color, *F*(1, 25) = 9.15, *p* = .006, ηG^2^ = .247, estimated *t*(25) = 3.025, 95% CI [3.83, 20.18], as well as several characteristics that did not differ between groups but are known to influence HCC, including age, body mass index, frequency of hair washing, hair treatment within the past 3 months, and current use of local glucocorticoids (inhalation), alcohol, nicotine, and hormonal contraceptives, *p*s of group difference = .009–.047. Within PTSD patients, HCC were not significantly correlated with total symptom severity, *r* = .16, *p* = .603, 95% CI [−.73, 55].

### Correlations Between Amygdala Reactivity and Negative Affective Stimuli

**Figure 2:**
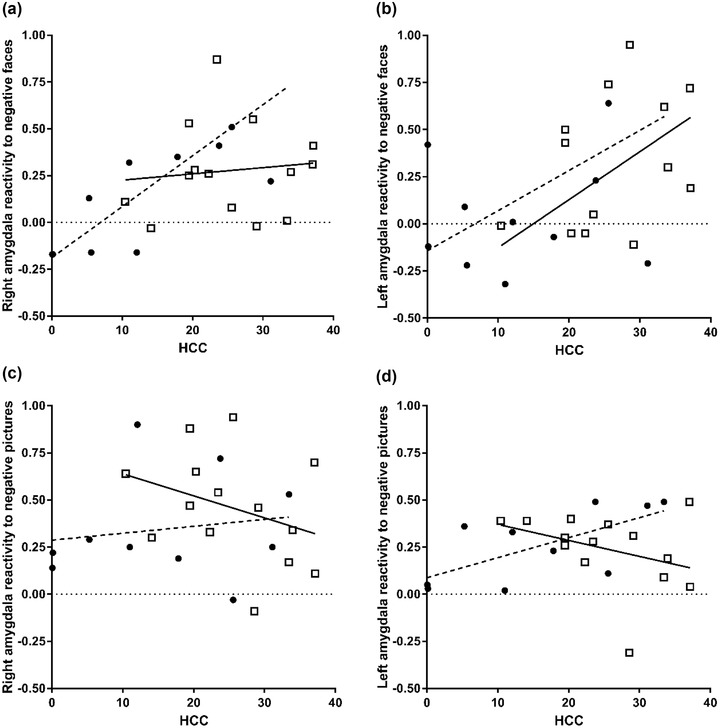
Scatterplots representing correlations between hair cortisol concentrations (HCC, in pg/mg) and contrast estimates of amygdala reactivity (arbitrary units) toward negative emotional faces (A = right; B = left) and negative affective pictures (C = right; D = left) in female trauma‐exposed police officers with (circles, dashed line) and without (squares, solid line) posttraumatic stress disorder (PTSD).

We investigated whether HCC was correlated with amygdala reactivity to negative affective stimuli in participants with PTSD and TCs and whether the magnitude of the correlation coefficient differed between groups. For right amygdala reactivity to negative affective faces compared to positive/neutral affective faces in the emotional face‐matching task, the magnitude of the correlation was significantly different between groups, *z* = 2.18, *p* = .030, 95% CI [0.06, 1.27], with a strong positive correlation with participants with PTSD, *r* = .81, *p* = .003, 95% CI [.55, .96]; and a nonsignificant correlation in TCs, *r* = .11, *p* = .715, 95% CI [−.36, .58] (Figure 2, Panel A). No differential correlation with HCC was observed for left amygdala reactivity to negative faces, *z* = ‐0.05, *p* = .963, 95% CI [−0.74, 0.63]. In both groups, HCC was not significantly associated with amygdala reactivity: ρ = .28, *p* = .401, 95% CI [−.41, .88] for the PTSD group and *r* = .50, *p* = .068, 95% CI [.07, −.78] for the TC group (Figure 2, Panel B). However, after controlling for the time of day when scanning took place, the magnitude of the correlation between HCC and left amygdala reactivity to negative faces was marginally increased for both groups: ρ = .49, *p* = .131, 95% CI [−.21, .94] for the PTSD group and *r* = .58, *p* = .036, 95% CI [.13, .86] for the TC group. Regarding passive viewing of the IAPS negative affective pictures compared to neutral pictures, no differential correlation with HCC was observed for right amygdala reactivity to negative affective pictures and no significant correlations were observed within groups, *z* = 1.05, *p* = .292, 95% CI [−0.40, 1.18], and *r* = .16, *p* = .658, 95% CI [−.41, .73] for the PTSD group and *r* = −.33, *p* = .243, 95% CI [−.84, .14] for the TC group (Figure 2, Panel C). The magnitude of the correlations between groups significantly differed for left amygdala reactivity, *z* = 2.40, *p* = .016, 95% CI [0.29, 1.48]. We observed a strong positive correlation within participants with PTSD, *r* = .66, *p* = .037, 95% CI [.15, .93], which was absent in the TC group, ρ = −.36, *p* = .203, 95% CI [−.80, .38] (Figure 2, Panel D). Partial correlations controlling for age and time of day the scanning took place did not alter magnitudes of observed correlations, other than those that have already been described. Amygdala reactivity to both negative faces and negative pictures was not significantly correlated to total symptom severity within participants with PTSD, *r*s = −.182–.298. Amygdala reactivity to the two types of negative stimuli was not significantly correlated, *r*s = −.279–.394.

## Discussion

Compared to female trauma‐exposed police officers without PTSD, we observed significantly lower HCC in female police officers with PTSD. This finding is in line with what was reported by Luo and colleagues (2012), who observed lower HCC in individuals with PTSD compared to TCs in a sample of female adolescent earthquake survivors several months after the earthquake; mean HCC in women with PTSD was also comparable to what was found in the current study. Our observed group difference is, however, in apparent contrast to results reported in two cross‐sectional studies, both of which found comparable HCC in trauma‐exposed individuals with and without PTSD in two predominantly female samples of individuals for whom a longer period of time had elapsed since trauma exposure (Schalinski et al., 2015; Steudte et al., 2013). Interestingly, however, in the only study to our knowledge that used similar immunoassays to those used in the current study (Russell et al., 2015), the observed mean HCC in individuals with PTSD was highly comparable to our observed mean HCC (Schalinski et al., 2015). Our finding is in apparent contrast with findings of higher HCC in individuals with PTSD compared to TCs in a mixed‐gender sample of internally displaced refugees of whom most individuals with PTSD, but not TCs, had pronounced ongoing distress (Steudte et al., 2011).

Steudte‐Schmiedgen, Kirschbaum, Alexander, and Stalder (2016) recently proposed a model on the course of trauma‐induced changes in cortisol output. It poses that cortisol output changes in a dose‐ and time‐dependent quadratic manner in response to trauma exposure, with initial elevated and subsequent chronically attenuated cortisol, independent of whether exposed individuals develop PTSD. This nonlinear association between trauma exposure and cortisol output may, in part, mediate the repeatedly reported dose–response association between increasing trauma load and increasing PTSD risk (but see also Kessler et al., 2017, for more recent findings on PTSD risk depending on type of previous trauma in combination with lifetime psychiatric history prior to index trauma). Notably, cross‐sectional studies that compare trauma‐exposed individuals with and without PTSD, including studies that assess HCC, typically report higher trauma exposure in individuals with PTSD. Although this fits with the earlier‐mentioned dose–response relationship between trauma load and PTSD risk, it may confound cross‐sectional investigations. Interestingly, our trauma‐exposed police officers without PTSD reported nominally higher work‐related trauma exposure than police officers with PTSD. Nevertheless, we still observed significantly lower HCC in participants with PTSD, which remained significant after controlling for work‐related trauma exposure. This suggests that in our noncivilian female sample, PTSD was associated with lower HCC independent of the effects of accumulating trauma exposure. As our female police officers with PTSD reported lower work‐related trauma exposure than their matched TCs, it is conceivable that they were more vulnerable to adverse mental health consequences of traumatic stress. Although our study had no longitudinal design, this fits with previously observed low pretrauma HCC (Steudte‐Schmiedgen et al., 2015) and high pretrauma glucocorticoid receptor function (van Zuiden et al., 2012) as predictive of PTSD symptom development in male soldiers. However, we only investigated the amount of work‐related traumatic event types participants had encountered and not time since exposure. This is relevant as the model proposed by Steudte‐Schmiedgen et al. (2016) describes elevated cortisol output in the acute period after trauma, prior to attenuated output. Additionally, the authors of a recent meta‐analysis found significant positive associations between ongoing chronic stress and HCC (Stalder et al., 2017). Thus, an alternative explanation is that our observed group difference reflects relatively elevated HCC in TCs due to ongoing or more recent exposure to work‐related traumatic stress rather than of a PTSD‐related attenuation. However, as the difference in HCC remained significant after controlling for current active police duty, we deem this alternative explanation less plausible. Additionally, the mean HCC observed in our TC group was comparable to the mean HCC reported in a mixed‐gender sample of TCs with little trauma exposure within the last year (Steudte et al., 2011).

Long‐term HCC is thought to be a relatively stable and reliable measure of long‐term cortisol output of the HPA axis (Staufenbiel et al., 2013). Therefore, the most intuitive interpretation is that our finding supports repeated findings on more acute measures of HPA axis function, indicating PTSD is associated with dysregulation of the HPA axis (Olff & van Zuiden, 2017). As previous studies have reported that HPA axis dysregulation may precede trauma exposure and PTSD development (van Zuiden, Kavelaars, Geuze, Olff, & Heijnen, 2013), chronic HPA axis dysregulation may be causally involved in pathophysiological processes underlying phenotypical expression and maintenance of some PTSD symptoms. Nevertheless, observed associations between HCC and PTSD status could also be influenced by PTSD risk factors associated with altered cortisol output, such as pain (Gaab et al., 2005), or health behaviors commonly associated with PTSD or general psychopathology, such as tobacco use (Olff et al., 2006) and decreased physical activity (Fekedulegn et al., 2018). However, we applied stringent inclusion and exclusion criteria and added several health behavior–related covariates to address potential confounders.

To further investigate whether and how HCC may be associated with the phenotypical expression of PTSD, we performed exploratory analyses to investigate whether HCC was associated with previously observed neural correlates of PTSD. We focused on amygdala reactivity to two types of negative affective stimuli—nontrauma related stimuli (faces) and a mixture of trauma‐related and non‐trauma‐related stimuli (pictures)—as authors of a recent meta‐analysis found higher bilateral amygdala reactivity in individuals with PTSD compared to TCs in response specifically to non‐trauma‐related stimuli (Stark et al., 2015). To facilitate comparison of results for both types of stimuli, we subtracted amygdala reactivity toward the neutral conditions from reactivity toward the negative conditions. Within participants with PTSD, but not in TCs, we observed a moderate‐to‐strong positive correlation between HCC and right amygdala reactivity to negative affective faces compared to neutral or positive faces. Furthermore, after correction for the time of day the scanning took place, a significant positive correlation between HCC and left amygdala reactivity to negative faces emerged for TCs, and a moderate positive correlation was observed for participants with PTSD, although this was not significant. We also observed a moderate‐to‐strong positive correlation between HCC and left amygdala reactivity to negative affective pictures compared to neutral pictures in participants with PTSD. Thus, lower HCC, in itself associated with PTSD status, was associated with diminished differentiation in amygdala reactivity between negative and neutral affective stimuli. This association was most pronounced for participants with PTSD.

If the peripheral findings related to HCC reflect persistent changes in circulating central cortisol, speculatively, the observed association between HCC and diminished differentiation in amygdala reactivity may be influenced by long‐term compensatory changes in central GR and/or MR signaling pathways, such as upregulated receptor expression and binding affinity or changes at the signaling route downstream of the receptor. Such changes may have occurred in the amygdala or other brain areas that modulate amygdala reactivity, changing amygdala reactivity to perceived negative stimuli. On the other hand, decreased HCC may result from high signaling in these receptor pathways, leading to reduced cortisol output by the HPA axis (Buckingham, 2006; De Bosscher, Van Craenenbroeck, Meijer, & Haegeman, 2008; de Quervain et al., 2017). Based on the current literature on GR and MR function in PTSD, neither of these two directions can be excluded.

In our larger study, we observed that, in contrast to TCs, police officers with PTSD did not show differentiation in amygdala reactivity to negative versus positive or neutral faces (Koch et al., 2016b). We hypothesized that participants with PTSD may have interpreted neutral faces in the neutral/positive faces condition as ambiguous stimuli signaling potential threat (i.e., increased attributed saliency), resulting in decreased differentiation in amygdala reactivity toward the two affective conditions. Such a lack of differentiation between negative and neutral stimuli may result from deficits in context processing (i.e., a diminished capacity to interpret the environment in a situation‐specific manner). A recent model addresses diminished context processing by hippocampal–prefrontal–thalamic brain circuitry modulating amygdala reactivity as key in the pathophysiology of PTSD and specifically intrusive symptomatology (Liberzon & Abelson, 2016). This brain circuitry is critically modulated by GR activation, with GRs mediating effects on contextual learning and memory consolidation depending on activation of beta‐adrenergic receptors in the basolateral amygdala (Quirarte, Roozendaal, & McGaugh, 1997). Clearly, although we observed that activation clusters for the negative pictures task extended posteriorly from the amygdala toward the hippocampus (Koch et al., 2018), the hypothesis that our observed correlation between HCC and decreased amygdala differentiation between negative and neutral emotional stimuli could be associated with context processing should be further investigated.

This study was the first, to our knowledge, to investigate associations between HCC and PTSD in a female noncivilian trauma‐exposed sample, but it had some important limitations. First, the study had a cross‐sectional design, and therefore, we cannot address questions of causality and directionality. In theory, relatively long hair strands provide the possibility to retrospectively assess HCC and therefore investigate HCC changes over time in relation to trauma and PTSD development. However, this seems more relevant when the demarcation of a single traumatic event in time is more apparent than it was in our sample. Also, the small sample size resulted in modest statistical power. Based on Bender and Lange's (2001) recommendation that multiple comparison corrections should not be applied for studies of an exploratory nature, we opted not to apply corrections for multiple testing in the correlational analyses. It should however be stressed that hypotheses derived from our exploratory correlational analyses warrant future confirmatory studies with adequate statistical power. Also, although affective faces are generally designated as non‐trauma‐related, the pictures task contained a mixture of non‐trauma‐related and likely trauma‐related stimuli that we could not further subdivide into separate conditions. Therefore, it remains to be investigated whether observed associations are specific to non‐trauma‐related stimuli or also hold for trauma‐related stimuli. Additionally, as we only included female police officers willing to participate in our pharmacological neuroimaging study, we cannot be certain that our findings generalize to the larger population of female police officers. Additionally, it remains to be investigated whether observed findings also extend to men. Furthermore, as mentioned, frequency and time since trauma exposure were not investigated nor was non‐work‐related trauma exposure during adulthood. We were also not able to include a trauma‐naive control group with similar demographic and work‐related characteristics as the included trauma‐exposed participants, as active police service for several years is generally associated with exposure to traumatic events in the line of duty. Together, these limitations precluded more detailed analyses of associations and directionality of causation.

In summary, we observed lower long‐term hair cortisol levels in female police officers with PTSD compared to trauma‐exposed female police officers without PTSD. Exploratory analyses indicated that lower HCC was associated with lower differentiation of amygdala reactivity between negative and neutral affective stimuli, which was more pronounced in participants with PTSD. Future studies should further investigate the associations between HCC, trauma, and PTSD, as well as associated neurobiological mechanisms.

## References

[jts22395-bib-0001] Ashburner, J. , Barnes, G. , Chen, C. , Daunizeau, J. , Flandin, G. , Friston, K. , … Philips, C. (2013). SPM8 Manual. London, UK: Welcome Trust Centre for Neuroimaging.

[jts22395-bib-0002] Bender, R. , & Lange, S. (2001). Adjusting for multiple testing–when and how? Journal of Clinical Epidemiology, 54, 343–349. 11297884 10.1016/s0895-4356(00)00314-0

[jts22395-bib-0003] Blake, D. D. , Weathers, F. W. , Nagy, L. M. , Kaloupek, D. G. , Gusman, F. D. , Charney, D. S. , & Keane, T. M. (1995). The development of a Clinician‐Administered PTSD Scale. Journal of Traumatic Stress, 8, 75. 7712061 10.1007/BF02105408

[jts22395-bib-0004] Bremner, J. D. , Bolus, R. , & Mayer, E. A. (2007). Psychometric properties of the Early Trauma Inventory‐Self Report. The Journal of Nervous and Mental Disease, 195, 211–218. 10.1097/01.nmd.0000243824.84651.6c 17468680 PMC3229091

[jts22395-bib-0005] Buckingham, J. C. (2006). Glucocorticoids: Exemplars of multi‐tasking. British Journal of Pharmacology, 147, S258–268. 10.1038/sj.bjp.0706456 16402112 PMC1760726

[jts22395-bib-0006] Bush, K. (1998). The AUDIT Alcohol Consumption Questions (AUDIT‐C): An effective brief screening test for problem drining. Archives of Internal Medicine, 158, 1789 10.1001/archinte.158.16.1789 9738608

[jts22395-bib-0007] Carlier, I. V. , & Gersons, B. P. (1992). Development of a scale for traumatic incidents in police officers. Psychiatria Fennica, 23, 59.

[jts22395-bib-0008] Cohen, J. (1977). Statistical power analysis for the behavioral sciences. New York City, NY: Routledge.

[jts22395-bib-0009] De Bosscher, K. , Van Craenenbroeck, K. , Meijer, O. C. , & Haegeman, G. (2008). Selective transrepression versus transactivation mechanisms by glucocorticoid receptor modulators in stress and immune systems. European Journal Pharmacology, 583, 290–302. 10.1016/j.ejphar.2007.11.076 18289525

[jts22395-bib-0010] de Quervain, D. , Schwabe, L. , & Roozendaal, B. (2017). Stress, glucocorticoids and memory: Implications for treating fear‐related disorders. Nature reviews.Neuroscience, 18, 7–19. 10.1038/nrn.2016.155 27881856

[jts22395-bib-0011] Diedenhofen, B. , & Musch, J. (2015). Cocor: A comprehensive solution for the statistical comparison of correlations. PloS one, 10, e0121945 10.1371/journal.pone.0121945 25835001 PMC4383486

[jts22395-bib-0012] Fekedulegn, D. , Innes, K. , Andrew, M. E. , Tinney‐Zara, C. , Charles, L. E. , Allison, P. , … Knox, S. S. (2018). Sleep quality and the cortisol awakening response (CAR) among law enforcement officers: The moderating role of leisure time physical activity. Psychoneuroendocrinology, 95, 158–169. 10.1016/j.psyneuen.2018.05.034 29864672 PMC6401560

[jts22395-bib-0013] First, M. B. , Spitzer, R. L. , Gibbon, M. , & Williams, J. B. (2002). Structured Clinical Interview for DSM‐IV Axis I Disorders (SCID‐I), Clinician version, aministration booklet. Washington, DC: American Psychiatric Publishing Inc.

[jts22395-bib-0014] Gaab, J. , Baumann, S. , Budnoik, A. , Gmunder, H. , Hottinger, N. , & Ehlert, U. (2005). Reduced reactivity and enhanced negative feedback sensitivity of the hypothalamus‐pituitary‐adrenal axis in chronic whiplash‐associated disorder. Pain, 119, 219–224. 10.1016/j.pain.2005.10.001 16298068

[jts22395-bib-0015] Hariri, A. R. , Tessitore, A. , Mattay, V. S. , Fera, F. , & Weinberger, D. R. (2002). The amygdala response to emotional stimuli: A comparison of faces and scenes. Neuroimage, 17, 317–323. 12482086 10.1006/nimg.2002.1179

[jts22395-bib-0016] Hayes, J. P. , Hayes, S. M. , & Mikedis, A. M. (2012). Quantitative meta‐analysis of neural activity in posttraumatic stress disorder. Biology of Mood & Anxiety Disorders, 2, 9 10.1186/2045-5380-2-9 22738125 PMC3430553

[jts22395-bib-0017] Hovens, J. E. , van der Ploeg, H. M. , Klaarenbeek, M. T. , Bramsen, I. , Schreuder, J. N. , & Rivero, V. V. (1994). The assessment of posttraumatic stress disorder with the Clinician Administered PTSD Scale: Dutch results. Journal of Clinical Psychology, 50, 325–340. 8071438 10.1002/1097-4679(199405)50:3<325::aid-jclp2270500304>3.0.co;2-m

[jts22395-bib-0018] Kessler, R. C. , Aguilar‐Gaxiola, S. , Alonso, J. , Bromet, E. J. , Gureje, O. , Karam, E. G. , … Zaslavsky, A. M. (2017). The associations of earlier trauma exposures and history of mental disorders with PTSD after subsequent traumas. Molecular Psychiatry, 23(9), 1–8 10.1038/mp.2017.194 PMC5858954 28924183

[jts22395-bib-0020] Koch, S. B. , van Zuiden, M. , Nawijn, L. , Frijling, J. L. , Veltman, D. J. , & Olff, M. (2016a). Aberrant resting‐state brain activity in posttraumatic stress disorder: A meta‐analysis and systematic review. Depression & Anxiety, 33, 592–605. 10.1002/da.22478 26918313

[jts22395-bib-0021] Koch, S. B. , van Zuiden, M. , Nawijn, L. , Frijling, J. L. , Veltman, D. J. , & Olff, M. (2016b). Intranasal oxytocin administration dampens amygdala reactivity towards emotional faces in male and female PTSD patients. Neuropsychopharmacology, 41, 1495–1504. 10.1038/npp.2015.299 26404844 PMC4832009

[jts22395-bib-0019] Koch, S. B. , van Zuiden, M. , Nawijn, L. , Frijling, J. L. , Veltman, D. J. , & Olff, M. (2018). Effects of intranasal oxytocin on distraction as emotion regulation strategy in patients with posttraumatic stress disorder (Advance online publication). European Neuropsychopharmacology. 10.1016/j.euroneuro.2018.12.002 30554861

[jts22395-bib-0022] Lang, P. J. , Bradley, M. M. , & Cuthbert, B. N. (2008). *International affective picture system (IAPS): Affective ratings of pictures and instruction manual* (A‐8). Gainesville University of Florida.

[jts22395-bib-0023] Liberzon, I. , & Abelson, J. L. (2016). Context processing and the neurobiology of posttraumatic stress disorder. Neuron, 92, 14 10.1016/j.neuron.2016.09.039 27710783 PMC5113735

[jts22395-bib-0024] Lobbestael, J. , Leurgans, M. , & Arntz, A. (2011). Inter‐rater reliability of the Structured Clinical Interview for *DSM‐IV* Axis I Disorders (SCID I) and Axis II Disorders (SCID II). Clinical Psychology and Psychotherapy, 18, 75–79. 10.1002/cpp.693 20309842

[jts22395-bib-0025] Luo, H. , Hu, X. , Liu, X. , Ma, X. , Guo, W. , Qiu, C. , … Li, T. (2012). Hair cortisol level as a biomarker for altered hypothalamic‐pituitary‐adrenal activity in female adolescents with posttraumatic stress disorder after the 2008 wenchuan earthquake. Biological Psychiatry, 72, 65–69. 10.1016/j.biopsych.2011.12.020 22305287

[jts22395-bib-0026] Manenschijn, L. , Koper, J. W. , Lamberts, S. W. , & van Rossum, E. F. (2011). Evaluation of a method to measure long term cortisol levels. Steroids, 76, 1032–1‐36. 10.1016/j.steroids.2011.04.005 21515299

[jts22395-bib-0027] McRae, K. , Hughes, B. , Chopra, S. , Gabrieli, J. D. , Gross, J. J. , & Ochsner, K. N. (2010). The neural bases of distraction and reappraisal. Journal of Cognitive Neuroscience, 22, 248–262. 10.1162/jocn.2009.21243 19400679 PMC4136451

[jts22395-bib-0028] Meewisse, M. L. , Reitsma, J. B. , de Vries, G. J. , Gersons, B. P. , & Olff, M. (2007). Cortisol and posttraumatic stress disorder in adults: Systematic review and meta‐analysis. The British Journal of Psychiatry, 191, 387–392. 10.1192/bjp.bp.106.024877 17978317

[jts22395-bib-0029] Mewes, R. , Reich, H. , Skoluda, N. , Seele, F. , & Nater, U. M. (2017). Elevated hair cortisol concentrations in recently fled asylum seekers in comparison to permanently settled immigrants and non‐immigrants. Transl Psychiatry, 7, e1051 10.1038/tp.2017.14 28267148 PMC5416663

[jts22395-bib-0030] Olejnik, S. , & Algina, J. (2003). Generalized eta and omega squared statistics: Measures of effect size for some common research designs. Psychological Methods, 8, 434–447. 10.1037/1082-989X.8.4.434 14664681

[jts22395-bib-0031] Olff, M. , Meewisse, M. L. , Kleber, R. J. , van der Velden, P. G. , Drogendijk, A. N. , van Amsterdam, J. G. , … Gersons, B. P. (2006). Tobacco usage interacts with postdisaster psychopathology on circadian salivary cortisol. International Journal of Psychophysiology, 59, 251–258. 10.1016/j.ijpsycho.2005.10.014 16387376

[jts22395-bib-0032] Olff, M. , & van Zuiden, M. (2017). Neuroendocrine and neuroimmune markers in PTSD: Pre, peri‐ and posttrauma glucocorticoid and inflammatory dysregulation. Current Opinion in Psychology, 14, 132–137. 10.1016/j.copsyc.2017.01.001 28813312

[jts22395-bib-0033] Pacella, M. L. , Hruska, B. , Steudte‐Schmiedgen, S. , George, R. L. , & Delahanty, D. L. (2017). The utility of hair cortisol concentrations in the prediction of PTSD symptoms following traumatic physical injury. Social Science & Medicine, 175, 228–234. 10.1016/j.socscimed.2016.12.046 28109728

[jts22395-bib-0034] Patel, R. , Spreng, R. N. , Shin, L. M. , & Girard, T. A. (2012). Neurocircuitry models of posttraumatic stress disorder and beyond: A meta‐analysis of functional neuroimaging studies. Neuroscience and Biobehavioral Reviews, 36, 2130–2142. 10.1016/j.neubiorev.2012.06.003 22766141

[jts22395-bib-0035] Quirarte, G. L. , Roozendaal, B. , & McGaugh, J. L. (1997). Glucocorticoid enhancement of memory storage involves noradrenergic activation in the basolateral amygdala. Proceedings of the National Academy of Sciences of the United States of America, 94, 14048. 9391150 10.1073/pnas.94.25.14048 PMC28430

[jts22395-bib-0036] Rademaker, A. R. , Vermetten, E. , Geuze, E. , Muilwijk, A. , & Kleber, R. J. (2008). Self‐reported early trauma as a predictor of adult personality: A study in a military sample. Journal of Clinical Psychology, 64, 863–875. 10.1002/jclp.20495 18428119

[jts22395-bib-0037] Russell, E. , Kirschbaum, C. , Laudenslager, M. L. , Stalder, T. , de Rijke, Y. , van Rossum, E. F. , … Koren, G. (2015). Toward standardization of hair cortisol measurement: Results of the first international interlaboratory round robin. Therapeutic Drug Monitoring, 37, 71–75. 10.1097/FTD.0000000000000148 25387254

[jts22395-bib-0038] Sartory, G. , Cwik, J. , Knuppertz, H. , Schurholt, B. , Lebens, M. , Seitz, R. J. , & Schulze, R. (2013). In search of the trauma memory: A meta‐analysis of functional neuroimaging studies of symptom provocation in posttraumatic stress disorder (PTSD). PloS one, 8, e58150 10.1371/journal.pone.0058150 23536785 PMC3607590

[jts22395-bib-0039] Schalinski, I. , Elbert, T. , Steudte‐Schmiedgen, S. , & Kirschbaum, C. (2015). The cortisol paradox of trauma‐related disorders: Lower phasic responses but higher tonic levels of cortisol are associated with sexual abuse in childhood. PloS one, 10, e0136921 10.1371/journal.pone.0136921 26317554 PMC4552952

[jts22395-bib-0040] Sheehan, D. V. , Lecrubier, Y. , Sheehan, K. H. , Amorim, P. , Janavs, J. , Weiller, E. , … Dunbar, G. C. (1998). The Mini‐International Neuropsychiatric Interview (M.I.N.I.): The development and validation of a structured diagnostic psychiatric interview for *DSM‐IV* and *ICD‐10* . The Journal of Clinical Psychiatry, 59(Suppl 20) 22. 9881538

[jts22395-bib-0041] Stalder, T. , Steudte‐Schmiedgen, S. , Alexander, N. , Klucken, T. , Vater, A. , Wichmann, S. , … Miller, R. (2017). Stress‐related and basic determinants of hair cortisol in humans: A meta‐analysis. Psychoneuroendocrinology, 77, 261–274. 10.1016/j.psyneuen.2016.12.017 28135674

[jts22395-bib-0042] Stark, E. A. , Parsons, C. E. , Van Hartevelt, T. J. , Charquero‐Ballester, M. , McManners, H. , Ehlers, A. , … Kringelbach, M. L. (2015). Posttraumatic stress influences the brain even in the absence of symptoms: A systematic, quantitative meta‐analysis of neuroimaging studies. Neuroscience and Biobehavioral Reviews, 56, 207–221. 10.1016/j.neubiorev.2015.07.007 26192104

[jts22395-bib-0043] Staufenbiel, S. M. , Penninx, B. W. , Spijker, A. T. , Elzinga, B. M. , & van Rossum, E. F. (2013). Hair cortisol, stress exposure, and mental health in humans: A systematic review. Psychoneuroendocrinology, 38, 1220–1235. 10.1016/j.psyneuen.2012.11.015 23253896

[jts22395-bib-0044] Steudte, S. , Kirschbaum, C. , Gao, W. , Alexander, N. , Schonfeld, S. , Hoyer, J. , & Stalder, T. (2013). Hair cortisol as a biomarker of traumatization in healthy individuals and posttraumatic stress disorder patients. Biological Psychiatry, 74, 639–646. 10.1016/j.biopsych.2013.03.011 23623187

[jts22395-bib-0045] Steudte, S. , Kolassa, I. T. , Stalder, T. , Pfeiffer, A. , Kirschbaum, C. , & Elbert, T. (2011). Increased cortisol concentrations in hair of severely traumatized Ugandan individuals with PTSD. Psychoneuroendocrinology, 36, 1193–1200. 10.1016/j.psyneuen.2011.02.012 21411229

[jts22395-bib-0046] Steudte‐Schmiedgen, S. , Kirschbaum, C. , Alexander, N. , & Stalder, T. (2016). An integrative model linking traumatization, cortisol dysregulation and posttraumatic stress disorder: Insight from recent hair cortisol findings. Neuroscience and Biobehavioral Reviews, 69, 124–135. 10.1016/j.neubiorev.2016.07.015 27443960

[jts22395-bib-0047] Steudte‐Schmiedgen, S. , Stalder, T. , Schonfeld, S. , Wittchen, H. U. , Trautmann, S. , Alexander, N. , … Kirschbaum, C. (2015). Hair cortisol concentrations and cortisol stress reactivity predict PTSD symptom increase after trauma exposure during military deployment. Psychoneuroendocrinology, 59, 123–133. 10.1016/j.psyneuen.2015.05.007 26072152

[jts22395-bib-0048] van Zuiden, M. , Geuze, E. , Willemen, H. L. , Vermetten, E. , Maas, M. , Amarouchi, K. , … Heijnen, C. J. (2012). Glucocorticoid receptor pathway components predict posttraumatic stress disorder symptom development: A prospective study. Biological Psychiatry, 71, 309–316. 10.1016/j.biopsych.2011.10.026 22137507

[jts22395-bib-0049] van Zuiden, M. , Kavelaars, A. , Geuze, E. , Olff, M. , & Heijnen, C. J. (2013). Predicting PTSD: Pre‐existing vulnerabilities in glucocorticoid‐signaling and implications for preventive interventions. Brain Behavior and Immunity, 30, 12–21. 10.1016/j.bbi.2012.08.015 22981834

[jts22395-bib-0050] Weathers, F. W. , Ruscio, A. M. , & Keane, T. M. (1999). Psychometric properties of nine scoring rules for the clinician‐administered posttraumatic stress disorder scale. Psychological Assessment, 11, 124–133.

